# Adipose Tissue Derived Multipotent Mesenchymal Stromal Cells Can Be Isolated Using Serum-free Media

**DOI:** 10.5812/ircmj.4506

**Published:** 2013-04-05

**Authors:** Iman Ahrari, Nima Purhabibi Zarandi, Mohsen Khosravi Maharlooei, Ahmad Monabati, Armin Attari, Sajjad Ahrari

**Affiliations:** 1 Student Research Committee, Shiraz University of Medical Sciences (SUMS), Shiraz, IR Iran; 2 Department of Pathology, Shiraz University of Medical Sciences (SUMS), Shiraz, IR Iran; 3 Department of Cardiovascular Medicine, Shiraz University of Medical Sciences (SUMS), Shiraz, IR Iran

**Keywords:** Mesenchymal Stem Cells, Cariel, Culture Medium, Cell Therapy

## Abstract

**Background:**

Mesenchymal stromal cells (MSCs) as multipotent cells with the capacity to be differentiated into several cell lineages are promising sources for cell therapy and tissue engineering nowadays. Today most of culturing media are supplemented with fetal bovine serum (FBS). But FBS containing culturing media may raise the possibility of zoonotic infections and immunological reactions in cell therapy conditions. Numerous investigations have been performed to assess the use of FBS-free culturing systems for bone marrow derived mesenchymal stromal cell isolation.

**Objectives:**

The present investigation aimed to assess the effect of serum-free media on growth and differentiating capacity of adipose tissue- derived MSCs.

**Materials and Methods:**

Approximately, 1cm3 surgically waste sterile adipose tissue was digested with collagenase-I leading to a single cell suspension. The isolated cells were cultured in Ultra Culture media supplemented with 2% Ultroser G. MSC’s isolation was confirmed with respect to morphology, flowcytometry, adipogenic and osteogenic differentiation potentials.

**Results:**

The isolated cells showed adherent spindle shaped morphology, expanded rapidly and revealed expected MSC flowcytometric characteristics; they were positive for CD73, CD90, CD105, CD44, CD166, CD44 and negative for hematopoietic antigen such as CD45, CD34 and CD14. They could also be differentiated successfully into osteoblast and adipocyte, being confirmed by using Alizarin Red and Oil red O staining, respectively.

**Conclusions:**

According to the results of the present study, it can be concluded that adipose derived MSCs can be cultured in serum-free media with no change in their differentiating capacity. This finding gives us a hope for future cell therapy studies and trials with little concern about zoonotic infections or immunological reaction.

## 1. Background

Multipotent stromal cells (MSCs), previously named mesenchymal stem cells (MSCs), are rare multipotent stem cells, residing mainly in the bone marrow (BM); however, they have also been isolated from other tissues like adipose tissue, umbilical cord (1, 2), dental pulp (3) and exfoliated deciduous teeth (4). These fibroblast-like cells can be differentiated by chemical or physiological stimulations. In vitro, culture-expanded MSCs are capable of differentiating into chondrocytes, adipocytes, osteoblasts (5), hepatocytes (6), myoblasts (7) and neural cells (8). Besides, MSCs’ multilineage differentiation ability, the ease of MSCs’ isolation accompanied by extensive capacity of expansion encouraged many scientists to utilize MSCs for tissue engineering as well as for gene therapy for a variety of congenital and acquired diseases (2). Cultured MSCs have been used in osteogenesis imperfecta (9), end stage liver disease (10), diabetic ulcers (11), and many other pathological conditions. Furthermore, due to established immunological properties (12), these cells have been used to promote engraftment and prevent or treat severe graft-versus-host disease (GVHD) in allogeneic stem cell transplantation (13) and prevent rejections in kidney transplant (14). Despite these findings, the role of these cells in oncology is being investigated (15). Since transplantation needs high numbers of MSCs, in vitro culturing and expansion is necessary. All previous transplantation studies have used cultures in a medium containing fetal bovine serum (FBS). Serum provides the growth factors and nutrients needed MSCs and also has the beneficial effects associated with antioxidant properties. Nevertheless, media containing animal proteins could cause different problems, such as transmission of zoonosis infectious agents or increasing the chance of allergic and immunologic reactions (16-18). This may end in antibody formation, non-engraftment or even rejection of the transplanted cells, especially if MSCs are transplanted for several times. This is supported by the observation that in one of the six patients with osteogenesis imperfecta who received expanded MSCs, an anti-FBS reaction was formed (9). Furthermore, anaphylactic or arthus-like immune reactions were reported in patients who received cells populated in FBS-supplemented medium (19), even causing arrhythmias after cellular cardioplasty (20). Tiny amounts of FBS will still remain on the cell surface, even after extensive washing and could cause toxicity in humans. It was shown that approximately 7–30 mg of FBS proteins can be carried by a single preparation of 108 MSCs expanded under standard condition in FBS-containing media (21). Finally the composition of FBS lots varies highly from lot to lot. Hence, the development of serum-free media is an obligation for clinical use of MSCs in wider applications. Currently, interest is growing in the use of FBS-free cultures for MSC isolation and expansion. In one relevant study, it was suggested that autologous serum with minimum concentration of 10% in the culture medium can be a good candidate (22). It is shown that life-threatening post-cellular cardiomyoplasty arrhythmias can be prevented by using autologous serum (AS) rather than FBS. Although most types of cellular therapy require large numbers of MSCs, large amounts of AS and subsequently large volumes of peripheral blood are difficult to obtain. Since allogeneic serum causes an arrest in the MSCs’ growth, using the pooled human serum cannot be helpful to overcome this issue (23). In a study, Claudia et al. has analyzed platelet lysate (PL) as a substitute for FBS. In comparison with FBS-containing media, they found a significant rise in both colony forming unit-fibroblast (CFU-F) and cumulative cell numbers after culture and expansion (24). To solve the problem of FBS hazards, some studies have also been conducted to culture bone marrow stromal cells by using human AB+ blood group sera instead of FBS (21, 25). But adult human serum does not have all of the essential growth factors for survival and expansion of stem cells. And since acquiring large volumes of human serum is difficult, it is not practically used for culturing cells, tissues and organs in vitro. Meuleman et al. (26) claimed an important practical advance in this field, using Ultra-culture medium with Ultroser G, a serum substitute to expand BM-MSCs. They were able to demonstrate preservation of multipotency together with faster growth of MSC’s than in traditional cultures. Different studies have investigated the results of BM derived MSCs culture in serum-free media but no data were found on adipose tissue-derived mesenchymal stem cells (AD-MSCs) culture. Adipose tissue is derived from the mesodermal germ layer, similar to BM, and contains a supportive stroma that can easily be isolated (27, 28). This stromal fraction of adipose tissue consists of a stromal vascular fraction containing the population of AD-MSCs (29). They need to be expanded in the culture before clinical use.

## 2. Objectives

Considering that the AD-MSCs can be obtained more easily and in higher numbers in comparison with BM, and also regarding the problems with FBS-substitutes mentioned above, we have designed this study to obtain MSCs from adipose tissue in a serum-free media.

## 3. Material and Method

### 3.1. Isolation and Culture of Adipose Tissue Stem Cells

AD-MSCs were isolated from lipoaspirates. The lipoaspirate was washed four times with equal volumes of Ca2‏- and Mg2‏-free PBS to remove the red blood cells and tissue debris. The washed tissue was resuspended in 150mL PBS containing 0.075% w/v collagenase A type I (Sigma, St. Louis, MO) and incubated on a shaker at 37ºC for 30 minutes. Collagenase activity was neutralized by adding an equal volume of αMEM (Invitrogen) with 10% fetal bovine serum (FBS; Gibco-RBL). After centrifugation at 3000g for 10 min, the cell pellets were resuspended in the culture medium and filtered through a 100-µm cell strainer (BD). The cells were precipitated (1200g, 10 min) and resuspended in 10mL of NH4Cl (160 mmol/L) for 10 min to lyse the remaining red blood cells. After centrifugation, the cell pellets were resuspended in Ultra-Culture medium (Cambrex) supplemented with 2% Ultroser G, a serum substitute (Pall BioSepra), 2 mm L-glutamine (GibcoBRL) and 1% antibiotic-antimycotic solution (GibcoBRL), added to tissue culture flasks, and cultured for 72 h at 37ºC in 5% CO2 and 90% humidity. Unattached cells and debris were then removed and fresh medium was added to the adherent cells. The cells were cultured to 80% confluence before being released with trypsin–EDTA and sub-cultured. The medium of the flasks changed every 3 days, and the cells were passaged for tree times.

### 3.2. Flowcytometry

To analyze the cell surface antigen expression, the cells from the third passage were harvested by 0.25% trypsin–EDTA. Trypsin was neutralized by FBS containing media and the isolated cells were washed twice with PBS. Then, the cells were incubated 30 min in dark environment following anti-human antibodies: CD90–fluorescein isothiocyanate (FITC), CD14-phycoerythrin (PE), CD34-FITC, CD166-PE, CD45-FITC, CD 44-FITC, CD105-PE (Serotec) and CD73-PE (Becton Dickinson). Mouse IgG1-PE and IgG2a-FITC (Serotec) were used as the isotype controls. The cells were analyzed by BD FACS-Calibur instrument.

### 3.3. Osteogenic Differentiation

For osteogenic differentiation, MSCs from the third passage were harvested by trypsin–EDTA 0.25%. The cells were cultured in NH-osteoDiff Medium (Miltenyi Biotec) at the density of 3×104 cells/ml in 2-chamber culture slides (Becton Dickinson) for 3 weeks according to the manufacturer’s guides and medium exchange was performed twice a week. To approve the differentiation, after appropriate morphological changes, the cells were analyzed by Alizarin red staining. Briefly, to perform the staining, the cells were washed once with PBS and fixed in methanol for 10 minutes. They were stained with the solution of 0.1M Alizarin Red (Sigma-Aldrich) in 25% Ammonia water for 24 hours and then the cells were washed once with distilled water.

### 3.4. Adipogenic Differentiation

For adipogenic differentiation, the cells from the third passage were harvested as mentioned above and were cultured in MesenCult medium (Stem Cell technologies) supplemented with 10% Adipogenic Stimulatory Supplements (Stem Cell technologies) at the density of 1.5×104 cells/ml in 2-chamber culture slides regarding the manufacturer’s guides. The cells were cultured for 3 weeks and half of the medium was exchanged only when the color of the media changed to yellow. As the cells showed appropriate morphological changes, they were analyzed with Oil-red O staining. Briefly, to perform the staining, the cells were fixed in 4% formalin containing 1% calcium chloride for an hour. Afterwards, the cells were stained with Oil-red O solution for 10-15 minutes and then counterstained with 70% ethanol for a minute and washed with distilled water. The solution was made with 0.05M of powder (Sigma-Aldrich) in 99% isopropranol which was diluted 3:2 with distilled water.

## 4. Results

### 4.1. Culture Characteristics

**Figure 1.**
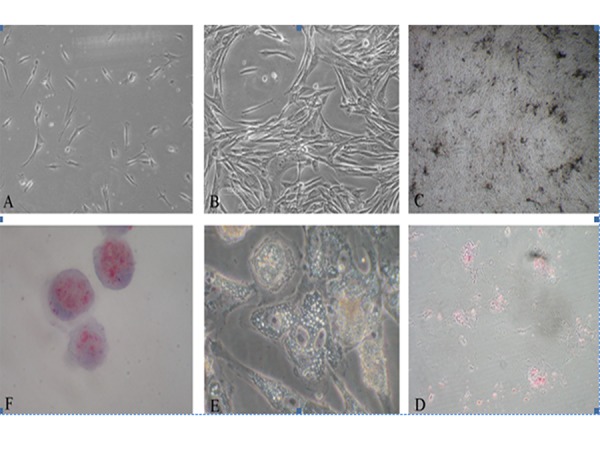
A) Primary seeding of the cells. B) Confluent cells. C) Osteoblast differentiated from MSC. D) Alizarin red staining of calcium deposits afterosteoblastic differentiation. E) Adipocyte differentiated from MSC. F) Lipid vacuoles stained with Oil red O after Adipocytic differentiation

Two days after primary cultivation, adherent spindle-shaped cells were detected (Figure 1A). Cells expanded rapidly in 5-6 days and showed homogenous morphology. These fibroblast-like cells formed homogenous colonies that confluented the flask within 15 days (Figure 1B). The rate of cell growth changed minimally after the increase in the number of passages but no morphological changes were noted.

### 4.2. Differentiation Assay

MSCs differentiation into osteoblast was initiated by detecting minimal depositions around the cells at the second week of seeding. Their differentiation into osteoblast lasted four weeks (Figure 1C). Mineralization was approved by Alizarin Red staining (Figure 1D). Adipogenic differentiation was proved both by morphological changes (Figure 1E) and Oil red O staining. One week after the cells were seeded in the adipogenic media, they showed small isolated vacuoles that increased in number and size for many times and all were stained by Oil red O (Figure 1F).

### 4.3. Flowcytometric Analysis

**Figure 2.**
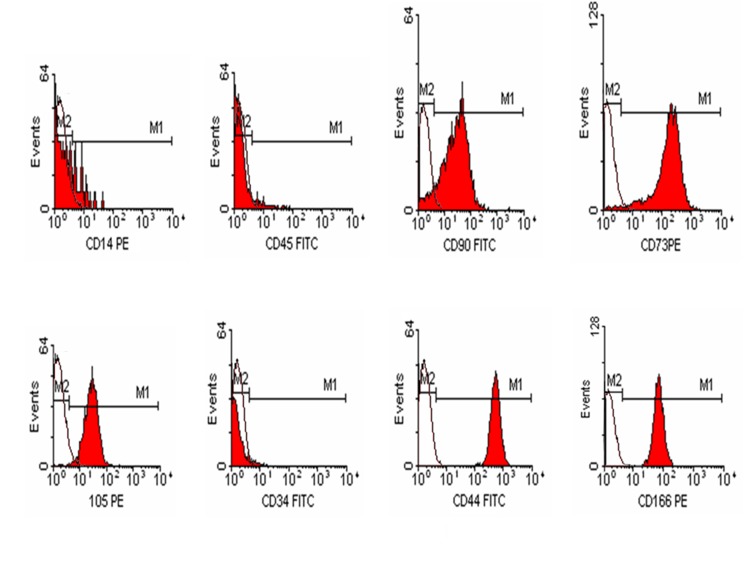
Immune Phenotypeof Adipose Tissue-Derived Fibroblast-Like Colony-Forming Cells

The isolated cells underwent flowcytometric analysis and were positive for CD73 (97.44%), CD105 (99.6%), CD44 (100%), CD166 (99.89%), CD90 (78.97%) and negative for hematopoietic antigen such as CD45 (1.87%), CD34 (1.54%) and CD14 (0.68%) (Figure 2).

## 5. Discussion

Cells isolated for cell therapy should fulfill the criteria proposed by the International Society for Cellular Therapy (30) to be considered as MSCs. These criteria are: 1) adherence to plastic; 2) 95% of the MSC population must express CD105, CD73 and CD90, as measured by flowcytometry. Additionally, these cells must lack expression (≤ 2% positive) of CD45, CD34, CD14 or CD11b, CD79a or CD19 and HLA class II; 3) these cells must be able to differentiate to osteoblasts, adipocytes or chondroblasts under in vitro differentiating conditions. MSCs with fulfilled criteria are currently used in clinical trials. This needs to meet such demands as safety, reproducibility and quality. It is also important to find ways for amplifying MSC in less time and define conditions to obtain standardized preparations of MSC with all their properties. Unfortunately, many of the applied culture media are unsuitable for clinical trial because they are based on animal products and consequently lead to the risk of transference of infective materials from animal to human on the table. In order to provide the safety, we must have a culture media which is free of animal-based substances and also save the multipotency and self-renewal ability of MSCs. In the present study, we first derived MSCs from the adipose tissues and expanded them in the serum-free media containing Ultra Culture (UC) medium supplemented with an artificial serum substitute, Ultroser-G S. Ultroser-G contains all nutrients needed for cell growth, i.e. binding proteins, growth factors, hormones and vitamins. On the other hand, its protein content is five times less than FBS. After the third passage, we determined the multipotency of these AD-MSCs by differentiating into adipocytes and osteoblasts with osteogenic and adipogenic media. This process was approved regarding the morphologic appearances of the cells cultured in osteogenic and adipogenic media and confirmed based on Alizarin Red and Oil Red O staining techniques, respectively. To define whether they are mesenchymal stromal cells based on their surface markers, we performed flowcytometry. Flowcytometric analysis revealed that MSCs cultured in the serum-free UC medium with Ultroser-G were positive for the cell surface markers known for MSC, such as CD44, CD166, CD73, CD90 and CD105. Hence, it can be concluded that MSCs cultured in the FBS-free media maintained their self-renewal and multipotency features which can then be differentiated to different cell lineages like adipocytes and osteocytes. Regarding these data, the cells we isolated in the serum-free media met the criteria of International Society of Cell therapy and were definitely MSCs. These data support the fact that isolation of AD-MSCs using a serum-free media is possible. It is promising to use the MSCs in clinical trials and treat different human diseases without the side effects that the animal serum such as FCS can introduce.
